# Antimicrobial Activity Against Phytopathogens and Inhibitory Activity on Solanine in Potatoes of the Endophytic Bacteria Isolated From Potato Tubers

**DOI:** 10.3389/fmicb.2020.570926

**Published:** 2020-11-17

**Authors:** Jia-Meng Liu, Shan-Shan Wang, Xu Zheng, Nuo Jin, Jia Lu, Ya-Tao Huang, Bei Fan, Feng-Zhong Wang

**Affiliations:** Key Laboratory of Agro-products Quality and Safety Control in Storage and Transport Process, Ministry of Agriculture and Rural Affairs/Institute of Food Science and Technology, Chinese Academy of Agricultural Sciences, Beijing, China

**Keywords:** potato (*Solanum tuberosum* L.), endophytes, antimicrobial activity, solanine, active component, Illumina-based analysis

## Abstract

As an important global crop, the potato (*Solanum tuberosum* L.) contains the endotoxin solanine that leads to human poisoning and major economic losses. Poisoning symptoms and even acute poisoning may occur when the content of solanine in potatoes exceeds 200 mg/kg. In addition, potatoes are susceptible to some pathogenic bacteria, including *Streptomyces scabies* and *Erwinia carotovora* subsp. *atroseptica* (Van Hall) dye, which can cause potato scab and potato blackleg disease, respectively. In this study, 37 culturable endophytic bacteria strains were obtained from potato tubers based on the culture-dependent method. Results indicated that nine strains showed antimicrobial activity against at least one pathogen by antimicrobial activity screening and 23 strains showed inhibitory activity on solanine in potato tubers. Among them, strain P-NA2-14 (*Bacillus megaterium* NBRC 15308^T^, 99.31%) showed not only better antimicrobial activity against both the two indicator pathogens, but also the best inhibitory activity on solanine, which was proved to be a potential biocontrol bacterium. Meanwhile, the relationship between the distribution of the endophytic bacterial community and the content of solanine in potato tubers was studied by Illumina-based analysis, indicating that the distribution of the endophytic bacterial community was obviously influenced by the content of solanine. The results showed a new insight into the relationship between plant secondary metabolites and endophytic bacteria in potato tubers and provided potential new technical support for the biological control of potato storage.

## Introduction

As one of the most important crops in the world, potato (*Solanum tuberosum* L.) ranks fourth after rice, wheat, and maize, and plays a significant role in the economy (Camire et al., 2009). According to the Food and Agriculture Organization of the United Nations (FAO) (FAOSTAT^1^), global potato production in the year 2018 was approximately 368 million tons. The top 3 producers of potatoes were as follows: China, mainland (90 million tons), India (48 million tons), and Ukraine (22 million tons).

As one of the most important crops for the food industry, potatoes need proper conditions for storage, or the content of glycoalkaloid solanine will increase. As a kind of toxic relative of steroidal glycoalkaloids (SGAs), solanine can cause great industrial losses (15% in China) due to breaking the dormancy period of potato tubers. Some research has found that SGAs are responsible for increasing the risk of brain, breast, lung, and thyroid cancers (Korpan et al., 2004; Grunenfelder et al., 2006). The solanine in potato tubers is mainly composed of α-solanine and α-chaconine, which can account for more than 95% of the content of solanine. It is generally accepted that the solanine content in potatoes is safe to eat when it does not exceed 100 mg/kg. Poisoning symptoms and even acute poisoning may occur when the content of solanine in potatoes exceeds 200 mg/kg (Koffi et al., 2017). The synthetic pathway of solanine in potatoes is unclear. There is no specific anti-solanine preparation in the market (Sawai et al., 2014). At present, the main methods to reduce the content of solanine are as follows: applying sprout suppressant (chlorpropham, CIPC), genetic modification, and controlling storage conditions. However, there are obvious deficiencies in both chemical and genetic modification methods. CIPC has been proven not to be safe for humans and the environment in recent years, and genetic modification has had limited success due to genetic instability, difficulty in operation, and a long production time (Smith and Bucher, 2012; Sawai et al., 2014). Therefore, it is particularly important to find a safer strategy to inhibit the increase of solanine content in potatoes. Meanwhile, potatoes are susceptible to many pathogenic bacteria, among them *Streptomyces scabies* and *Erwinia carotovora* subsp. *atroseptica* (Van Hall) dye are important pathogens of potatoes, causing potato scab and potato blackleg disease, respectively. These diseases can lead to heavy economic loss to the yield and quality of potatoes not only in the field but also during storage (Ali et al., 2013; Alkhazindar et al., 2016).

As an important determinant of promoting plant growth and maintaining plant health, endophytes have received substantial attention in recent years (Turner et al., 2013; Miliute et al., 2015). Numerous studies have been done to highlight the ability of endophytes to influence the important traits of host plants such as disease resistance, plant growth, water retention, abiotic stress tolerance, and the synthesis of plant growth-promoting hormones (Köberl et al., 2013; Huang W. et al., 2018). The large amount of novel and bioactive secondary metabolites produced by endophytes, which are not only beneficial to the host plants but also play a significant role in the pharmaceutical, agricultural, and food industries. So far, the studies on the endophytic bacteria of potato tubers are mainly focused on promoting host plant growth and inhibiting pathogenic bacteria, but there are few studies on the correlation between endophytic bacteria and the secondary metabolites of host plants (Pavlo et al., 2011; Da et al., 2012; Pageni et al., 2014).

The endophytic bacteria of potato tubers were isolated and identified using a culture-dependent method in this study. Then the strains with inhibitory activity on solanine and antibacterial activity were screened. Meanwhile, the relationship between the distribution of the endophytic bacterial community and the content of solanine in potato tubers was studied using Illumina-based analysis. This study provides potential new technical support for decreasing solanine during potato storage. At the same time, the results revealed a new comprehension on the relationship between endophytic bacteria and plant secondary metabolites of potato tubers.

## Materials and Methods

### Sterilizing Potato Samples

Potato samples (Crop Strains: DaXiYang) were collected from Ulanqab, Inner Mongolia Autonomous Region, China. All samples were stored at 4°C in cold storage. After the unpeeled potato samples were surface-sterilized according to the method in Wang et al. (2019), 200 μL of the final water rinse was plated on 11 different media (Supplementary Table 1). A total of 1% plant extracts of potato tubers were added to the 11 different media to offer natural growth conditions for the potential endophytic bacteria in plant tissues. The method of adding plant extracts of potato tubers to medium was as follow: 10 g potato tubers were squeezed in 100 mL of ultrapure water and filtered with four to six layers of gauze. A total of 10 mL plant extracts of potato tubers were added to the 1 L medium. Then the medium was sterilized at 121°C for 15 min. The different media were incubated at 28°C for 2 weeks to confirm the sterilization process was successful.

### Isolation and Identification of Endophytic Bacteria

#### Isolation of Endophytic Bacteria From Potato Tubers

The sterilized potato tubers were cut by sterile surgical scissors into 0.5 ∼ 1.0 cm segments. The segments were then placed onto 11 different media and then incubated at 28°C until the bacterial colonies emerged.

Single-colony isolation was repeated at least three times by use of the YIM38 agar medium for purification of the endophytic bacteria isolates. Finally, the selected isolates were stored in 50% (v/v) glycerol [bacterial fluid: glycerol (50%) = 1:1, v/v] at −80°C. Criterion for strain nomenclature was as follow: P-medium type-strain number.

#### Identification of Endophytic Bacteria

The endophytic bacteria DNA was extracted by the methods of Zhou et al. (2010). Then the 16S rDNA sequences of endophytic bacterial isolates were amplified with universal primers 27F (5′-AGAGTTTGATCCTGGCTCAG-3′) and 1492R (5′-TACGGCTACCTTGTTACGACTT-3′) by a thermocycler PCR system (Applied Biosystems, Singapore) (Mao et al., 2012). The PCR reaction was performed according to the method of Wang et al. (2019). The PCR products were checked on 1.2% agarose gel and were sequenced using the dideoxy chain-termination method. The two overlapping sequence reads of each endophytic bacterial isolate were assembled by the SeqMan of DNASTAR.Lasergene.7.1, then compared by the 16S-based ID of EzBioCloud (2018) database, and submitted to NCBI Genbank. The phylogenetic tree was constructed by the neighbor-joining method through the MEGA7.0 software.

### Screening for Antimicrobial Activity of Endophytic Bacteria Against Phytopathogen

#### Collection of Solvent Extract of Endophytic Bacteria

Both the selected endophytic bacteria and actinomycetes were incubated in 150 mL YIM38 liquid medium at 28°C and 200 rpm, actinomycetes were shaken for 7 days and bacteria were shaken for 2 days. The supernatant was collected after the fermentation broth was centrifuged at 9,500 rpm for 20 min at 4°C, and mixed with an equal volume of ethyl acetate. The ethyl acetate layer was collected using a separating funnel and dried by the rotary evaporator (RE-52AA; YARONG, China) at 38°C. Then, the extracts were redissolved in 1 mL of methanol and stored at −20°C.

#### Screening for Antimicrobial Activity

*Erwinia carotovora* subsp. *atroseptica* (Van Hall) dye (ACCC 19901) and *S. scabies* (ACCC 41024) were used to study the antimicrobial activities of the solvent extract. These phytopathogens were obtained from the Agricultural Culture Collection of China (ACCC). The antimicrobial activity was tested by the Kirby-Bauer test.

The test plates were prepared as follows: *E. carotovora* subsp. *atroseptica* (Van Hall) dye was cultured in 50 mL of the beef extract-peptone (BP) liquid medium with continuous shaking at 28°C and 200 rpm for 24 h, while *S. scabies* was cultured in 50 mL of the Kohlberg’s No. 1 (KN1) liquid medium under the same conditions. Then, 10 mL of the fermentation broth of the two phytopathogens were added to 100 mL of the BP and KN1 agar medium, respectively, and mixed gently. The mixed medium was poured on a petri dish slowly, which was used as the test plate. Sterilized paper disks (diameter, 6 mm) were saturated with the solvent extract (20 μL) and placed onto the test plates. An equivalent volume of methanol was used as negative control. Plates were then incubated at 28°C for 20 h. The diameter of the inhibition zones was measured to evaluate the effect of antimicrobial activity using an electronic digital caliper (0–150 mm).

### Inhibitory Activity on Solanine in Potato Tubers

An *in vivo* inhibition assay was designed to screen the single colony of selected isolates which could decrease the solanine in potato tubers. The single colony of endophytes were inoculated into 50 mL YIM38 liquid medium and incubated at 28°C with continuous shaking at 200 rpm for 24 h. The 20 mL of the inoculum (10%, v/v) was transferred into 200 mL fermentation medium (YIM38 liquid medium) and incubated at 28°C with continuous shaking at 200 rpm for 48 h, while the single colony of endophytic actinomycetes were shaken for 7 days under the same conditions. The unpeeled potato tubers were washed with running tap water and sprayed evenly with fermentation broth until the surface of the tubers were wet. An equivalent volume of YIM38 liquid medium without endophytic bacterial isolates was used as negative control, while an equivalent volume of chlorpropham (CIPC, a common potato bud suppressor on the market) served as positive control. Potato tubers were then stored under light at 28°C for 7 days. The content of solanine in potato tubers were determined by ultra-high performance liquid chromatography coupled to triple-quadrupole mass spectrometry (UPLC-QQQ-MS/MS) according to the method in Dai et al. (2017). There were six parallel samples in each group.

### Extraction of Active Components of Strain P-NA2-14

The strain P-NA2-14A was selected for further separation of active ingredients as it showed the highest inhibitory activity. It was fermented according to the method in Section “Inhibitory Activity on Solanine in Potato Tubers” and centrifuged at 9,500 rpm for 20 min at 4°C, the supernatant and pellet of the culture broth were collected separately. The supernatant was mixed with an equal volume of ethyl acetate, and the sediment was soaked in 15 mL of acetone for 24 h. Then, the potato tubers were sprayed evenly with 7 different groups of ingredients, including pure culture medium (F); ethyl acetate (EA); acetone (Ace); the acetone extracts of the sediment of strain P-NA2-14 (JT); the strain P-NA2-14 supernatant fermented broth (JY); the ethyl acetate extracts of the supernatant of strain P-NA2-14 (P-NA2-14EA); and the aqueous phase extract of the supernatant of strain P-NA2-14 (P-NA2-14A), to screen out the ingredient which had the most obvious inhibitory effect on solanine and would be used for further study.

The component P-NA2-14A that showed the highest inhibition on solanine was extracted using the column chromatography with AB-8 macroporous adsorption resin which was packed by the wet packing method. The P-NA2-14A was placed into macroporous adsorption resin and soaked for 2 h. Then the active components were eluted with different proportions of acetone-water (90, 80, 70, 60, 40, 20, and 10%, v/v) under a flow rate of 27 mL/min and collected in conical flasks every 10 min, respectively. The unpeeled potato tubers were sprayed evenly with the each collected active component. An equivalent volume of acetone and CIPC served as negative and positive controls, respectively. No less than three simultaneous experiments were performed for each component. Potato tubers were then stored under light for 7 days at 28°C. The content of solanine in potato tubers were determined using the same method as Section “Inhibitory Activity on Solanine in Potato Tubers.”

### Illumina-Based Analysis of Endophytic Bacteria

The unpeeled potato tubers were sprayed evenly with the different aqueous phase extract of the supernatant of strain P-NA2-14 until the surface of the tubers were wet, including Group CK (treated with blank medium, the content of solanine was 273 mg/kg); Group L (treated with the active ingredient 40–20, the content of solanine was 163 mg/kg); and Group H (treated with the active ingredient 20–30, the content of solanine was 344 mg/kg). Potato tubers were stored under light for 7 days at 28°C and then surface-sterilized as described in Section “Potato Samples Sterilizing.”

Microbial DNA was extracted from potato tubers using the E.Z.N.A.^®^ Soil DNA Kit (Omega Bio-tek, Norcross, GA, United States). The V4–V5 region of the bacteria 16S rDNA sequences were amplified by PCR using primers 338F (5′-ACTCCTACGGGAGGCAGCAG-3′) and 806R (5′-GGACTACHVGGGTWTCTAAT-3′) (Huang X. et al., 2018). The PCR amplification procedure is shown in Supplementary Table 2. The PCR products were extracted from 2% agarose gels and further purified using the AxyPrep DNA Gel Extraction Kit (Axygen Biosciences, Union City, CA, United States) and quantified using QuantiFluor^TM^-ST (Promega, United States). Purified amplicons were pooled in equimolar and paired-end sequenced (2 × 250) on an Illumina MiSeq platform. The raw reads were deposited into the Sequence Read Archive (SRA) of the NCBI database. Raw fastq files were quality-filtered by Trimmomatic (v0.30) and merged by FLASH (v1.2.7) (Bolger et al., 2014). Operational Units (OTUs) were clustered with 97% similarity cutoff using UPARSE (2020) (v7.1) and chimeric sequences were identified and removed using UCHIME (2020) (Edgar et al., 2011). The taxonomy of each 16S rRNA gene sequence was analyzed by RDP Classifier (2020) (v2.2) against the SILVA (2020) 16S rRNA (Release132) database using a confidence threshold of 70%.

Operational Unit levels with 97% similarity were selected for Alpha diversity analyses using the Mothur (2020) (version v.1.30.1) and the diversity indices included Shannon (The Shannon diversity index), Simpson (The Simpson diversity index), Sobs (The observed richness), Chao (The Chao1 estimator), Ace (The ACE estimator), and Coverage (The Good’s coverage) (Kim et al., 2017; Yang et al., 2017). Rarefaction curves were plotted to determine the abundance of communities and sequencing data of the samples. A Venn diagram was used to show unique and shared OTUs using the stats package in R (Deng et al., 2018). *Principal Component Analysis* (PCA) and Hierarchical clustering analysis on the genus level were also determined using the vegan package in R, respectively to detect whether there was a significant difference between the samples.

## Results

### Isolation and Identification of Endophytic Bacteria

**FIGURE 1:**
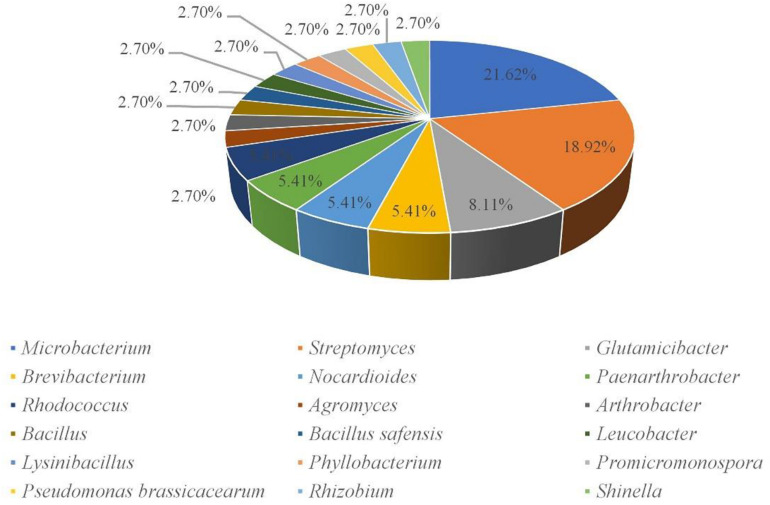
Percent of cultivable endophytes on the genus level.

**FIGURE 2:**
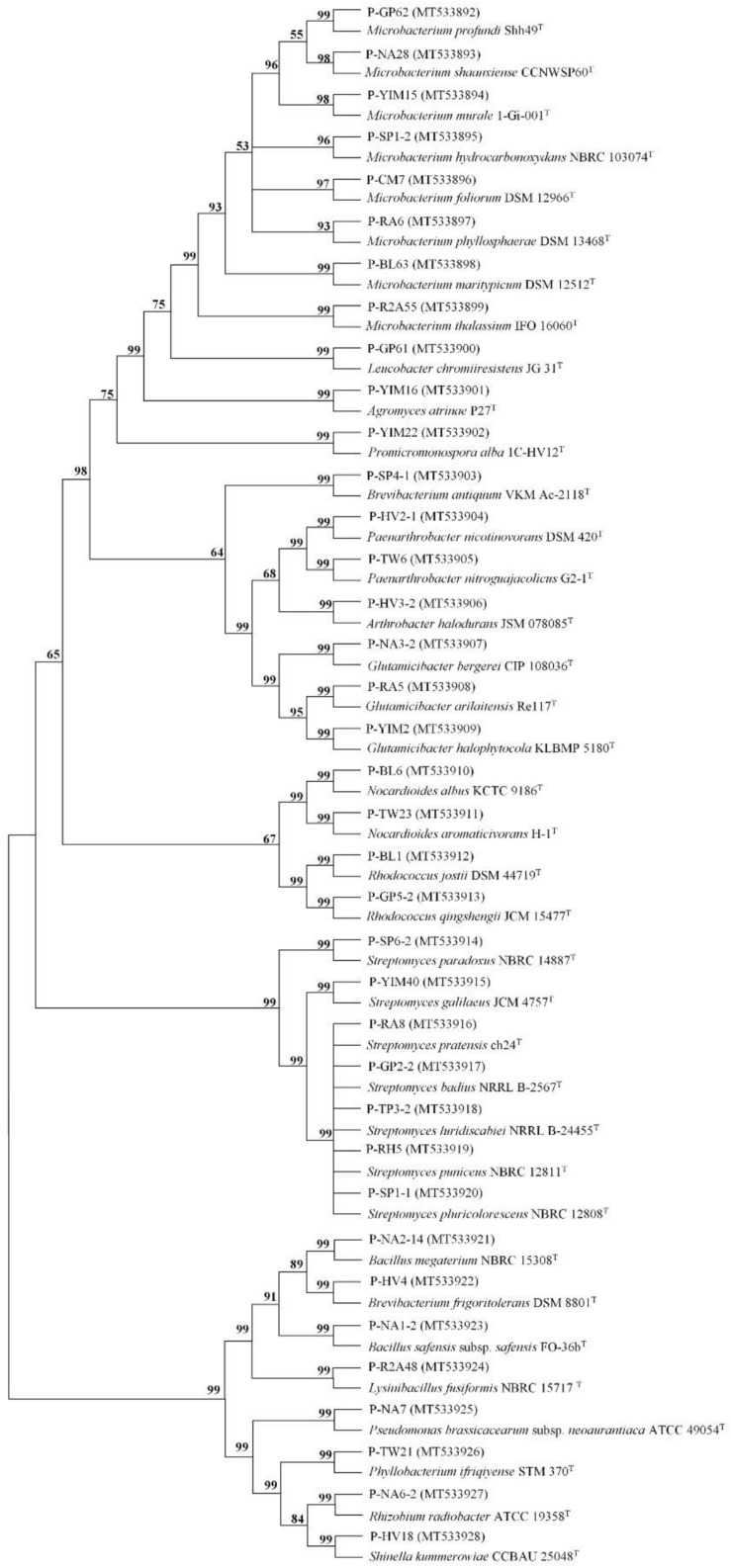
The tree of the 37 strains identified in this study. The tree was constructed with the neighbor-joining method with 1,000 bootstrap replicates. (GenBank accession numbers were given in parentheses. Strain name: P-medium type-strain number).

A total of 160 cultivable endophytic bacteria isolates were isolated from the sterilized potato tubers. All the isolated strains were identified by 16S rRNA gene sequence analysis and compared by the EzBioCloud (2018) database. All of the endophytic bacteria were classified into 3 different phyla (Firmicutes, Proteobacteria, and Actinobacteria), 18 genera, and 37 species. The 16S rRNA gene sequences of the 37 strains were submitted to the NCBI GenBank with the Accession Numbers (MT533892-MT533928) and dominated by *Microbacterium* (21.62%) and *Streptomyces* (18.92%) (Figure 1). A phylogenetic tree was constructed for these 37 isolates to demonstrate their evolutionary phylogenetic relationship (Figure 2). In terms of genus diversity, the phylogenetic tree in Figure 2 showed that *Microbacterium* was dominant harboring eight strains, followed by *Streptomyces* with seven strains, and *Glutamicibacter* with three strains. Each of the genus *Paenarthrobacter*, *Nocardioides*, *Rhodococcus*, and *Brevibacterium* harbored two strains. Meanwhile, other genera harbored one strain.

### Antimicrobial Activity of Endophytic Bacteria Against Phytopathogens

The 37 strains were tested for their antimicrobial activity against *E. carotovora* subsp. *atroseptica* (Van Hall) dye (ACCC 19901) and *S. scabies* (ACCC 41024) using the Kirby-Bauer method. Nine strains showed antimicrobial activity against at least one pathogen. These nine strains belonged to eight different genera, namely *Shinella*, *Bacillus*, *Rhodococcus*, *Phyllobacterium*, *Streptomyces*, *Rhizobium*, *Microbacterium*, and *Brevibacterium*. All the nine strains showed antimicrobial activity against the *E. carotovora* subsp. *atroseptica* (Van Hall) dye in this study, while only three strains showed antimicrobial activity against the *S. scabies* (Table 1). The extracts of the strain P-HV18 (*S. kummerowiae* CCBAU 25048^T^, 98.74%) showed the highest antimicrobial activity against the two phytopathogens. The pictures of the antimicrobial activity of some strains against phytopathogens are shown in Supplementary Figure 1.

**TABLE 1:** Endophytic strains which showed antimicrobial activity against at least one of the two phytopathogens.

Strain number	GenBank accession number	Closest species in 16S rRNA gene sequences database	Similarity (%)	Inhibition to *Erwinia carotovora* subsp. *atroseptica* (Van Hall) dye (mm)	Inhibition to *Streptomyces scabies* (mm)
P-HV18	MT533928	*Shinella kummerowiae* CCBAU 25048^T^	98.74	10.5	22.5
P-NA2-14	MT533921	*Bacillus megaterium* NBRC 15308^T^	99.31	8.2	16.8
P-GP5-2	MT533913	*Rhodococcus qingshengii* JCM 15477^T^	100	8.5	18.8
P-TW21	MT533926	*Phyllobacterium ifriqiyense* STM 370^T^	99.49	12.6	–
P-GP2-2	MT533917	*Streptomyces badius* NRRL B-2567^T^	99.93	10.6	–
P-NA6-2	MT533927	*Rhizobium radiobacter* ATCC 19358^T^	100	10.2	–
P-YIM40	MT533915	*Streptomyces galilaeus* JCM 4757^T^	99.85	9.0	–
P-CM7	MT533896	*Microbacterium foliorum* DSM 12966^T^	99.34	7.6	–
P-HV4	MT533922	*Brevibacterium frigoritolerans* DSM 8801^T^	100	7.4	–

### Inhibitory Activity on Solanine in Potato Tubers

**FIGURE 3:**
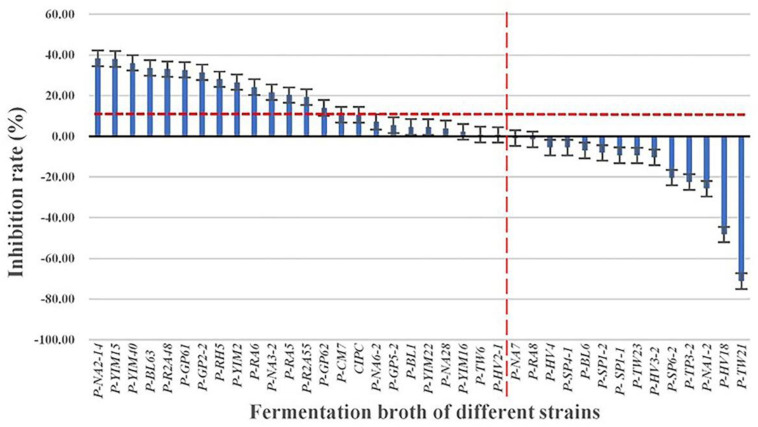
Inhibition effect of fermentation broth of 37 strains on the solanine of potato tubers. Three replicates for each condition. Error bars represent standard error (*n* = 3). [CIPC: a common potato bud suppressor on the market, which served as positive control. An equivalent volume of YIM38 liquid medium without endophytic bacterial isolates was used as negative control. Inhibition rate (%) = A–B/B. A: The content of solanine in different conditions; B: the content of solanine in negative control].

The solanine content of potato tubers were analyzed by UPLC-QQQ-MS/MS after being treated with fermentation broth of the 37 strains (Figure 3). Compared with the control group (YIM38 liquid medium without endophytic bacterial isolates), 23 strains showed inhibition of solanine in potato tubers, while 14 strains increased the content of solanine. As a common potato bud suppressor in the market, CIPC (positive control) can inhibit the content of solanine in potato tubers with an inhibition rate of 10.52%. Interestingly, there were 15 strains that showed a better inhibition activity than CIPC. The activity was as follows: P-NA2-14 (38.37%), P-YIM15 (38.07%), P-YIM40 (36.11%), P-BL63 (33.63%), P-R2A48 (33%), P-GP61 (32.67%), P-GP2-2 (31.45%), P-RH5 (28.05%), P-YIM2 (26.59%), P-RA6 (24.19%), P-NA3-2 (21.62%), P-RA5 (20.30%), P-R2A55 (19.24%), P-GP62 (13.94%), and P-CM (10.58%) (Table 2). Among them, strain P-NA2-14 (*B. megaterium* NBRC 15308^T^, 99.31%) showed the highest inhibition on the content of solanine and was selected for further study. It is worth noting that strain P-TW21 (*Phyllobacterium ifriqiyense* STM 370^T^, 99.49%) could make the content of solanine in potato tuber obviously increase, which also needed further study.

**TABLE 2:** The inhibitory rate on solanine and the similarity rate with the closest species in the 16S rRNA gene sequence database of the 37 strains.

Strain number	Closest species in 16S rRNA gene sequences database	Similarity (%)	Inhibition rate on solanine (%)
CIPC			10.52
P-NA2-14	*Bacillus megaterium* NBRC 15308^T^	99.31	38.37
P-YIM15	*Microbacterium murale* 1-Gi-001^T^	99.77	38.07
P-YIM40	*Streptomyces galilaeus* JCM 4757^T^	99.85	36.11
P-BL63	*Microbacterium maritypicum* DSM 12512^T^	99.36	33.63
P-R2A48	*Lysinibacillus fusiformis* NBRC 15717^T^	99.86	33.04
P-GP61	*Leucobacter chromiiresistens* JG 31^T^	99.44	32.67
P-GP2-2	*Streptomyces badius* NRRL B-2567^T^	99.93	31.45
P-RH5	*Streptomyces puniceus* NBRC 12811^T^	99.86	28.05
P-YIM2	*Glutamicibacter halophytocola* KLBMP 5180^T^	98.77	26.59
P-RA6	*Microbacterium phyllosphaerae* DSM 13468^T^	99.30	24.19
P-NA3-2	*Glutamicibacter bergerei* CIP 108036^T^	99.24	21.62
P-RA5	*Glutamicibacter arilaitensis* Re117^T^	99.12	20.30
P-R2A55	*Microbacterium thalassium* IFO 16060^T^	98.17	19.24
P-GP62	*Microbacterium profundi* Shh49^T^	98.04	13.94
P-CM7	*Microbacterium foliorum* DSM 12966^T^	99.34	10.58
P-NA6-2	*Rhizobium radiobacter* ATCC 19358^T^	100	7.07
P-GP5-2	*Rhodococcus qingshengii* JCM 15477^T^	100	5.39
P-BL1	*Rhodococcus jostii* DSM 44719^T^	99.19	4.62
P-YIM22	*Promicromonospora alba* 1C-HV12^T^	99.64	4.45
P-NA28	*Microbacterium shaanxiense* CCNWSP60^T^	99.93	3.88
P-YIM16	*Agromyces atrinae* P27^T^	100	2.27
P-TW6	*Paenarthrobacter nitroguajacolicus* G2-1^T^	100	0.88
P-HV2-1	*Paenarthrobacter nicotinovorans* DSM 420^T^	100	0.70
P-NA7	*Pseudomonas brassicacearum* subsp. *neoaurantiaca* ATCC 49054^T^	99.49	−0.90
P-RA8	*Streptomyces pratensis* ch24^T^	100	−1.47
P-HV4	*Brevibacterium frigoritolerans* DSM 8801^T^	100	−5.52
P-SP4-1	*Brevibacterium antiquum* VKM Ac-2118^T^	98.91	−5.52
P-BL6	*Nocardioides albus* KCTC 9186^T^	99.70	−7.00
P-SP1-2	*Microbacterium hydrocarbonoxydans* NBRC 103074^T^	99.78	−8.16
P-SP1-1	*Streptomyces pluricolorescens* NBRC 12808^T^	99.78	−9.36
P-TW23	*Nocardioides aromaticivorans* H-1^T^	99.71	−9.40
P-HV3-2	*Arthrobacter halodurans* JSM 078085^T^	99.63	−10.32
P-SP6-2	*Streptomyces paradoxus* NBRC 14887^T^	99.85	−20.32
P-TP3-2	*Streptomyces luridiscabiei* NRRL B-24455^T^	99.93	−22.45
P-NA1-2	*Bacillus safensis* subsp. *safensis* FO-36b^T^	99.86	−25.74
P-HV18	*Shinella kummerowiae* CCBAU 25048^T^	98.74	−48.28
P-TW21	*Phyllobacterium ifriqiyense* STM 370^T^	99.49	−71.20

*(The 16S rRNA gene sequence of strain P-R2A55 and P-GP62 showed a lower similarity rate with the closest species in the 16S rRNA gene sequence database, indicating that they are potential new species).*

### Extraction of Active Components of Strain P-NA2-14

**FIGURE 4:**
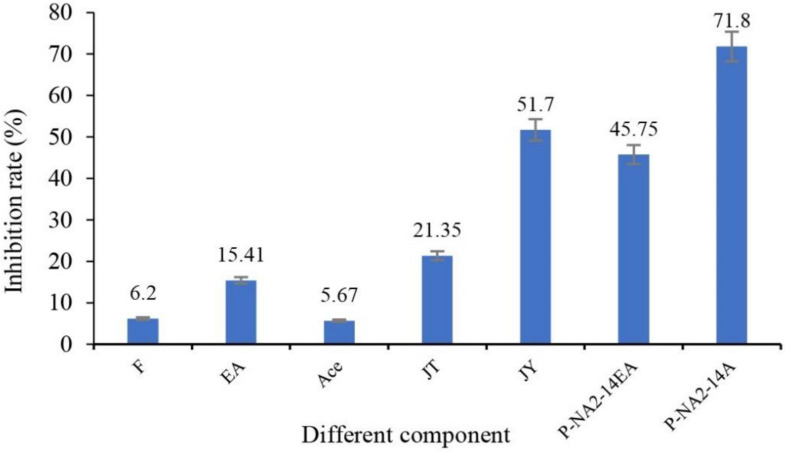
Effects of different components on the inhibition of solanine in potato tubers. Three replicates for each condition. Error bars represent standard error (*n* = 3). (F: potato tubers were sprayed evenly with the pure culture medium; EA: potato tubers were sprayed evenly with the ethyl acetate; Ace: potato tubers were sprayed evenly with the acetone; JT: potato tubers were sprayed evenly with acetone extracts of the sediment of strain P-NA2-14; JY: potato tubers were sprayed evenly with the strain P-NA2-14 supernatant fermented broth; P-NA2-14EA: potato tubers were sprayed evenly with the ethyl acetate extracts of the supernatant of strain P-NA2-14; and P-NA2-14A: potato tubers were sprayed evenly with the aqueous phase extract of the supernatant of strain P-NA2-14).

After being treated with seven different groups of ingredients, the inhibition rates of solanine in potatoes are shown in Figure 4. Among them, P-NA2-14A (71.8%), JY (51.7%), P-NA2-14EA (45.75%), and JT (21.35%) showed better inhibition on solanine than others, while P-NA2-14A (71.8%) showed the highest inhibitory activity.

**FIGURE 5:**
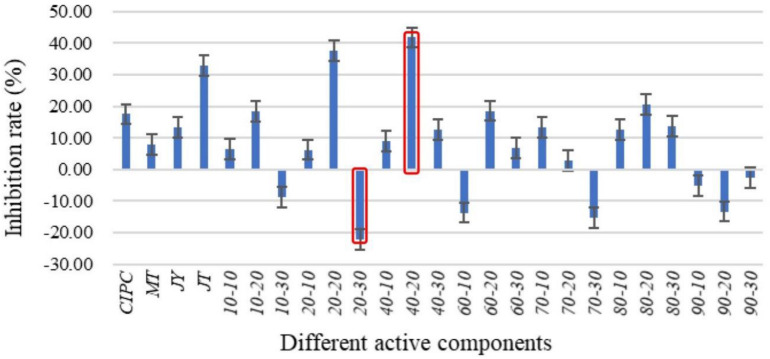
Effects of different active components of P-NA2-14A on the inhibition of solanine in potato tubers. Three replicates for each condition. Error bars represent standard error (*n* = 3). (Group CIPC was treated with chlorpropham; Group MT was treated with acetone; Group JY was treated with strain P-NA2-14 supernatant fermented broth; Group JT was treated with strain P-NA2-14 cells; Groups 10–10, 10–20, and 10–30 were treated with 10% acetone extract collected in 0–10, 10–20, and 20–30 min, respectively; Groups 20–10, 20–20, and 20–30 were treated with 20% acetone extract collected in 0–10, 10–20, and 20–30 min, respectively; Groups 40–10, 40–20, and 40–30 were treated with 40% acetone extract collected in 0–10, 10–20, and 20–30 min, respectively; Groups 60–10, 60–20, and 60–30 were treated with 60% acetone extract collected in 0–10, 10–20, and 20–30 min, respectively; Groups 70–10, 70–20, and 70–30 were treated with 70% acetone extract collected in 0–10, 10–20, and 20–30 min, respectively; Groups 80–10, 80–20, and 80–30 were treated with 80% acetone extract collected in 0–10, 10–20, and 20–30 min, respectively; and Groups 90–10, 90–20, and 90–30 were treated with 90% acetone extract collected in 0–10, 10–20, and 20–30 min, respectively).

P-NA2-14A was selected for further separation of the active ingredients since it had the highest inhibitory activity. P-NA2-14A was separated into different components after the preliminary screening (Figure 5). Meanwhile, the active components which showed inhibition to solanine were obtained. Among the 21 different components, 14 active ingredients showed inhibitory activity on solanine, while 7 active ingredients increased the content of solanine compared to the control group. As for the 21 active ingredients, group 40–20 (inhibition rates of solanine was 41.82%) showed the best inhibitory activity, while group 20–30 (inhibition rates of solanine was −22.32%) could obviously increase the content of solanine in potato tubers. Therefore, group 40–20 and group 20–30 were selected for Illumina-based analysis in order to study the influence of those compositions on the distribution of endophytic bacteria in potato tubers. The 21 active ingredients were extracted from the fermentation products of the strain P-NA2-14A at different times by using acetone solution of different proportions. The analysis of the specific composition of each active ingredient was carried out next in our study.

### Diversity of Endophytic Bacteria Based on Illumina-Based Analysis

#### Quality Control and Analysis of Sequencing Data

**FIGURE 6:**
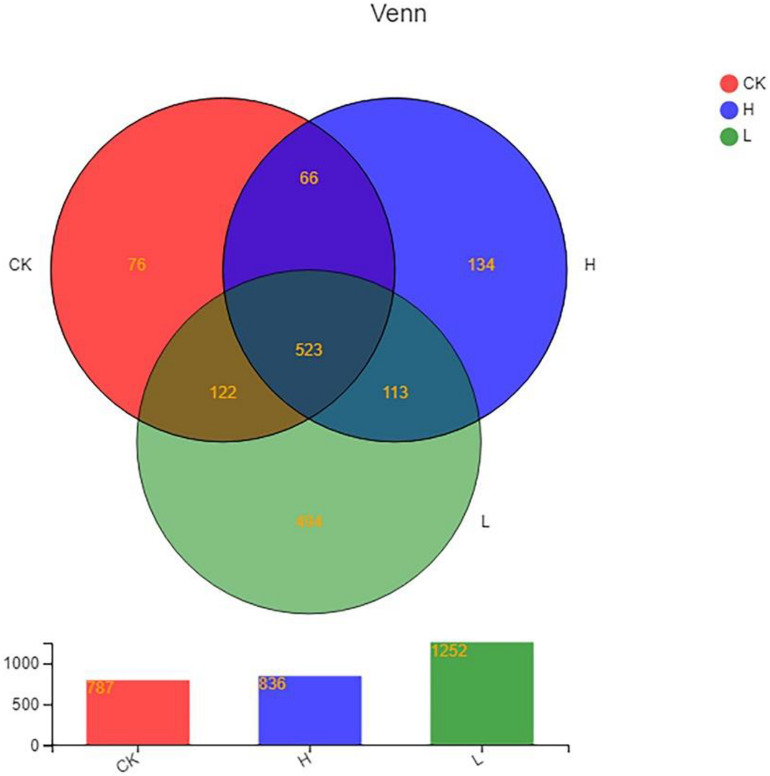
Venn diagram showing the number of OTUs shared and unique among different samples. (Group CK: the unpeeled potato tubers were treated with blank medium, the content of solanine was 273 mg/kg; Group L: the unpeeled potato tubers were treated with the active ingredient 40–20, the content of solanine was 163 mg/kg; and Group H: the unpeeled potato tubers were treated with the active ingredient 20–30, the content of solanine was 344 mg/kg).

A total of 614,140 reads and 1,528 OTUs were obtained after clustering at a 97% similarity level from the 3 groups: Group CK, Group L, and Group H (three parallel in each group). As the rarefaction curves tended to approach saturation, this showed that the sequencing work was relatively comprehensive in covering the bacterial diversity and the selected sequence data adequately reflected the bacterial abundance of the samples (Supplementary Figure 2). The distributions of endophytic microbial composition on the OTU level in different samples were also detected using the Alpha-diversity analyses including Shannon, Sobs, Simpson, Chao, Ace, and Coverage to estimate the endophytic community complexity (Table 3). Sobs, Chao, and Ace indexes reflected the community richness. Shannon and Simpson indexes reflected the community diversity, while Coverage indexes reflected the community coverage (Kim et al., 2017; Yang et al., 2017). The community abundance of endophytes on OTU level in Group L was higher than the other two groups. The differences in OTU abundance of the 3 groups is also shown in the Venn diagram. The OTU abundance of endophytic bacterial was the highest in Group L with the lowest solanine content. The three groups shared 523 OTUs, while they each harbored 76 (Group CK), 494 (Group L), and 134 (Group H) unique OTUs, respectively (Figure 6).

**TABLE 3:** Number of OTUs and alpha-diversity analyses of endophytes on OTU level in potato tubers.

Group	Sample	Reads	OTUs	Alpha diversity					
				Shannon	Sobs	Simpson	Chao	Ace	Coverage
CK	CK1	46399	501	3.4911	501	0.1006	554.1148	550.2843	99.75%
	CK2	38258	567	3.3406	567	0.1434	637.4430	637.7981	99.63%
	CK3	44813	477	3.0259	477	0.1543	496.5283	497.8110	99.88%
L	L1	43670	262	4.9789	609	0.0187	620.1111	616.2608	99.93%
	L2	64119	356	3.9379	355	0.0477	371.7308	371.7174	99.94%
	L3	54890	973	5.1033	969	0.0336	976.0455	977.6770	99.93%
H	H1	52218	565	3.9488	570	0.0823	621.0923	623.4289	99.81%
	H2	61226	533	4.2384	533	0.0649	557.4737	545.3226	99.94%
	H3	69951	430	4.1559	433	0.0707	441.5714	440.2407	99.97%

*(Group CK: the unpeeled potato tubers were treated with blank medium, the content of solanine was 273 mg/kg; Group L: the unpeeled potato tubers were treated with the active ingredient 40–20, the content of solanine was 163 mg/kg; and Group H: the unpeeled potato tubers were treated with the active ingredient 20–30, the content of solanine was 344 mg/kg).*

#### Composition and Diversity of Endophytic Bacterial Community in Different Groups

**FIGURE 7:**
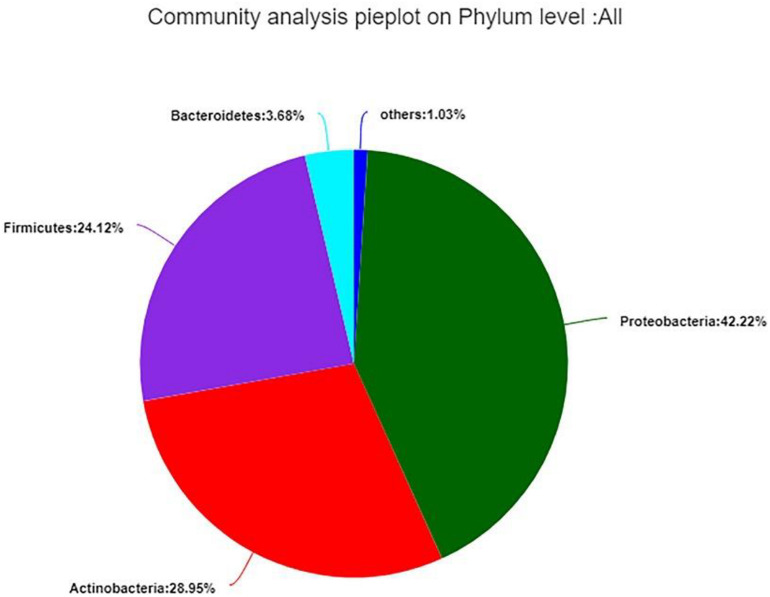
Composition and relative abundance of endophytic bacterial in different samples on phylum level. Phyla making up less than 1% of total composition in the samples were classified as “other.”

The 99.99% of bacterial sequences were classified from phylum to genus and 0.01% were unclassified. The valid sequences were classified into 24 different phyla, 47 classes, 122 orders, 280 families, 574 genera, and 942 species. In total, *Proteobacteria* (42.22%) was the dominant phylum, following by *Actinobacteria* (28.95%), *Firmicutes* (24.12%), and *Bacteroidetes* (3.68%), the remaining 1.03% contained 20 very low-abundant phyla (Figure 7). While the distribution of endophytic bacterial was different in each group.

**FIGURE 8:**
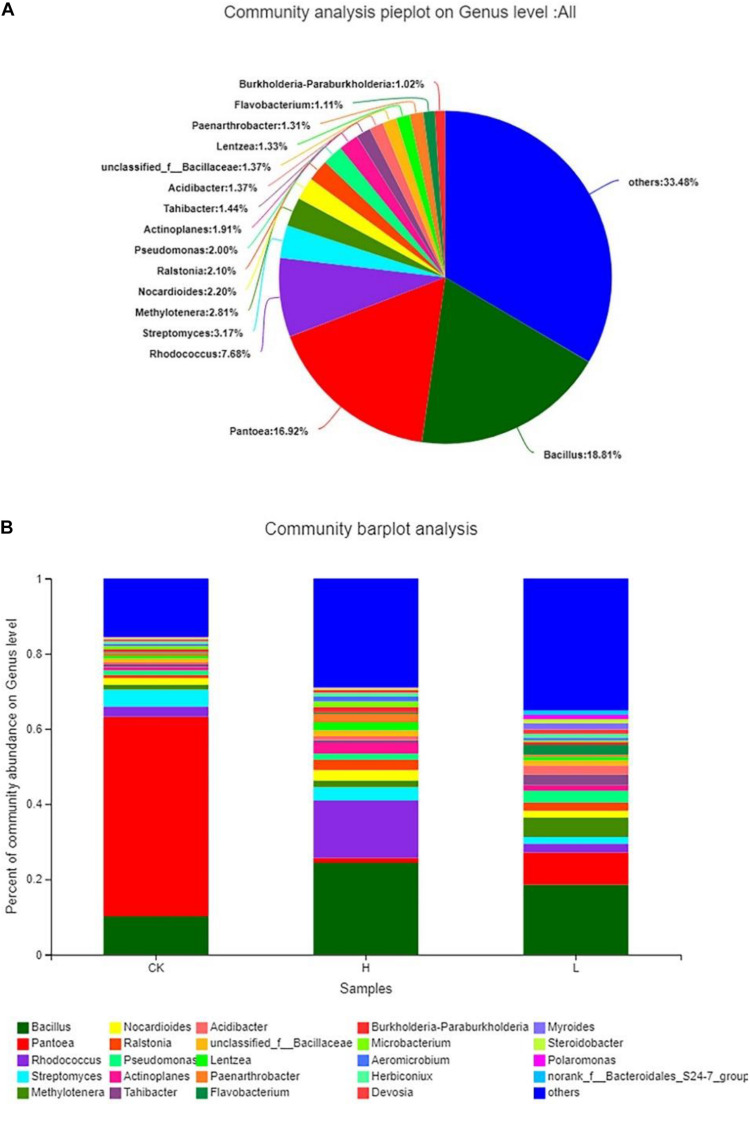
Composition and relative abundance of endophytic bacterial on the genus level. **(A)** Community analysis pie plot on the genus level of the culture-independent endophytic bacteria on the whole. **(B)** Composition and relative abundance of endophytic bacterial in different samples on the genus level. The color of the column represents the different genera, and the length of the column represents the proportion size of the genus. Sequences that could not be classified into any known group were assigned as “unclassified.” Genera making up less than 1% of total composition in each sample were classified as “other.” (Group CK: the unpeeled potato tubers were treated with blank medium, the content of solanine was 273 mg/kg; Group L: the unpeeled potato tubers were treated with the active ingredient 40–20, the content of solanine was 163 mg/kg; and Group H: the unpeeled potato tubers were treated with the active ingredient 20–30, the content of solanine was 344 mg/kg).

The results showed that the distribution of each dominant genus also varied among the three groups. Overall, *Bacillus* (18.81%) accounted for the highest proportion of the 3 groups, followed by *Pantoea* (16.92%), and *Rhodococcus* (7.68%), while there were 33.48% genera with below 1% abundance (Figure 8A). Meanwhile, Group L had the highest community abundance, followed by Group H, and Group CK had lowest community abundance (Figure 8B). However, the dominant genera were different from each other. There were 18 genera with over 1% abundance in Group CK and the dominant genera were *Pantoea* (53.06%) and *Bacillus* (10.16%). There were 18 genera with over 1% abundance in Group L and the dominant genera were *Bacillus* (18.61%) and *Pantoea* (8.55%). There were 20 genera with over 1% abundance in Group H and the dominant genus was *Bacillus* (24.40%) and *Rhodococcus* (15.26%). The abundance of the same genus was significantly different among the three groups. For example, although *Pantoea* was detected in all three groups, it had the highest proportion in the CK Group, accounting for 53.06%, following by Group L (8.55%) and Group H (1.28%). *Actinoplanes* (2.94%), *Lentzea* (2.10%), and *Nocardioides* (2.77%) showed a higher proportion in Group H than the other two groups. *Acidibacter* (2.46%), *Methylotenera* (5.13%), *Pseudomonas* (3.09%), and *Tahibacter* (2.86%) showed a higher proportion in Group L than the other two groups. *Myroides* (1.70%) and *Polaromonas* (1.13%) were only detected in Group L (Table 4).

**TABLE 4:** Comparison of percentage (%) of the metagenome sequences affiliated with the dominant bacterial genera (average abundance >1%) for the three groups.

Genus	CK	L	H
*Acidibacter*	0.55	2.46	0.96
*Actinoplanes*	0.90	1.44	2.94
*Aeromicrobium*	0.62	–	1.31
*Bacillus*	10.16	18.61	24.40
*Burkholderia-Paraburkholderia*	0.69	–	1.43
*Devosia*	0.51	1.21	0.70
*Flavobacterium*	–	2.64	0.34
*Herbiconiux*	0.64	0.98	1.01
*Lentzea*	0.68	0.89	2.10
*Myroides*	–	1.70	–
*Methylotenera*	1.24	5.13	1.84
*Microbacterium*	0.92	–	1.47
*Nocardioides*	1.79	1.82	2.77
*Pantoea*	53.06	8.55	1.28
*Paenarthrobacter*	0.73	–	2.31
*Pseudomonas*	1.24	3.09	1.55
*Polaromonas*	–	1.13	–
*Rhodococcus*	2.76	2.34	15.26
*Ralstonia*	0.85	2.24	2.77
*Steroidobacter*	–	1.03	0.46
*Streptomyces*	4.47	1.77	3.53
*Tahibacter*	0.71	2.86	0.70
Other	15.47	35.04	28.93

*(Group CK: the unpeeled potato tubers were treated with blank medium, the content of solanine was 273 mg/kg; Group L: the unpeeled potato tubers were treated with the active ingredient 40–20, the content of solanine was 163 mg/kg; and Group H: the unpeeled potato tubers were treated with the active ingredient 20–30, the content of solanine was 344 mg/kg).*

**FIGURE 9:**
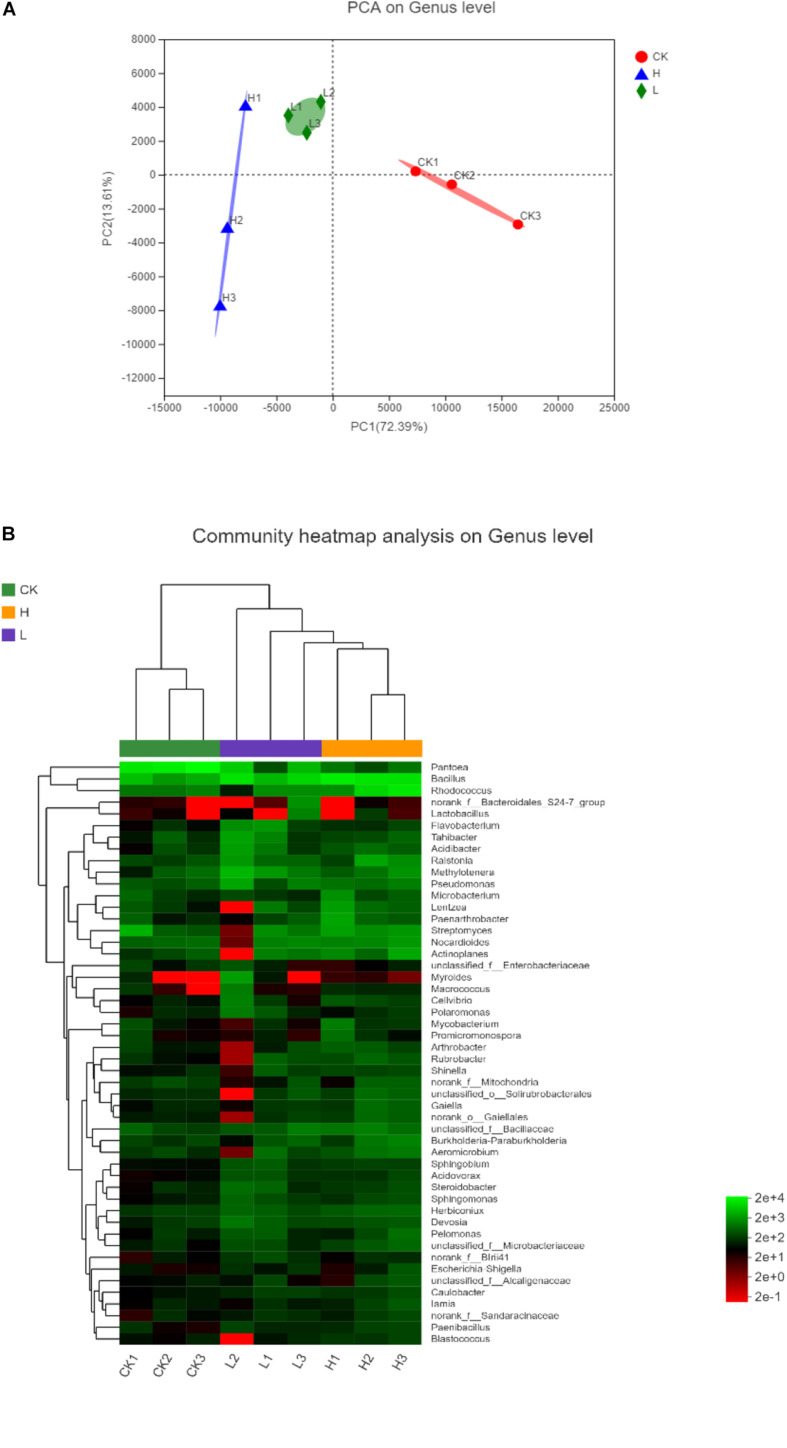
The differences among the samples in different samples on genus level. **(A)** *Principal Component Analysis* (PCA) illustrates differences between bacterial communities in the three groups. **(B)** Heatmap of the top 50 most abundant genera in bacterial communities detected in the three groups. Dendrograms for hierarchical cluster analysis grouping genera and sample locations were shown at the left and at the top, respectively. (Group CK: the unpeeled potato tubers were treated with blank medium, the content of solanine was 273 mg/kg; Group L: the unpeeled potato tubers were treated with the active ingredient 40–20, the content of solanine was 163 mg/kg; and Group H: the unpeeled potato tubers were treated with the active ingredient 20–30, the content of solanine was 344 mg/kg).

The significance of inter-group differences among the samples in the grouping were tested using the *Principal Component Analysis* (PCA) to conduct the linear discrimination and classification modeling for grouping samples. As shown in PCA analysis, the proportion of PC1 (72.39%) and PC2 (13.61%) indicated that the bacterial community composition of potato tubers with different solanine contents was significantly different at the genus level (Figure 9A). The similarity of endophytic bacteria among samples was also detected by Hierarchical clustering analysis. As shown in the heatmap, it was found that the endophytic community of Group H and Group L obviously clustered, while the endophytic community of Group CK was significantly different from the other two groups. Since all potato samples were stored in the same conditions, it indicated that the solanine content could be an important factor to shape the community composition of endophytic bacteria in potato tubers (Figure 9B).

## Discussion

As an important determinant of promoting plant growth and maintaining plant health, endophytic bacteria have received substantial attention in recent years (Turner et al., 2013; Miliute et al., 2015). Endophytic bacteria of plants may produce a range of different metabolites *in vitro* and within host plant tissue, which are rich sources of secondary metabolites with biological activity (Brader et al., 2014; Miliute et al., 2015). The bioactive secondary metabolites such as alkaloids, terpenoids, phenols, antibiotics, and anti-cancerous compounds produced by endophytic bacteria have been applied in agricultural, industrial, and medical fields (Christina et al., 2013; Gao et al., 2015; Liu et al., 2015; Yamada et al., 2015; Hashem et al., 2016). *Bacillus stratosphericus* LW-03 isolated from the bulbs of *Lilium wardii* exhibited multiple plant growth-promoting traits, including the production of indole-3-acetic acid (IAA), organic acids, and siderophores (Khan et al., 2020). The fermentation broth of *Aranicola proteolyticus*, *Bacillus licheniformis*, *Bacillus thuringiensis*, *Bacillus cereus*, and *Serratia liquefaciens* isolated from *Pinellia ternata* (Thunb.) berit contained alkaloids that were similar to those of the host plants (Liu et al., 2015). In the recent 3 years, at least 128 new endophytic bacteria species of plants were published, including four new genera: *Roseitalea*, *Allobranchiibius*, *Mangrovicella*, and *Oceaniradius* (Ai et al., 2017; Hyeon et al., 2017; Li et al., 2018; Jeong et al., 2019). Among them, three new endophytic bacteria strains were isolated from potatoes, including *Pectobacterium polaris* sp. nov NIBIO1006^T^ (=DSM 105255^T^ = NCPPB 4611^T^), *Pectobacterium parvum* s0421^T^ (CFBP 8630^T^ = LMG 30828^T^), and *Sphingobacterium solani* sp. nov. MLS-26-JM13-11^T^ (=ACCC 60057^T^ = JCM 32274^T^) (Dees et al., 2017; Niu et al., 2018; Pasanen et al., 2020). These statistics indicated that, as the treasure troves of new microorganism resources, endophytic bacteria should be given great attention, and it is worthy of further studies on the biological activities of endophytic bacteria such as antimicrobial activity, growth-promoting and bioactive secondary metabolites (Massimiliano et al., 2020).

In this study, 160 cultivable endophytic bacteria were isolated and assigned into 3 different phyla, 18 genera, and 37 species. As can be seen from Table 2, all the 18 genera identified in this study have been reported in previous studies (Sturz and Matheson, 1996; Yuan et al., 2002; Ramríez-Bahena et al., 2015a, b). The phylogenetic tree in Figure 2 showed that strain P-GP62 and P-R2A55 have a close relationship with the *Microbacterium* genus isolates, while the 16S rRNA gene sequence of strain P-GP62 (*Microbacterium profundi* Shh49^T^, 98.04%) and P-R2A55 (*Microbacterium thalassium* IFO 16060^T^, 98.17%) showed a lower similarity rate with the closest species in the 16S rRNA gene sequence database, indicating that they were potential new species, which should be studied further (Figure 2 and Table 4). As the culturable bacteria were part of the largest, most active bacteria in plant samples, culture-dependent and culture-independent techniques need to be combined to identify some rare and promising beneficial endophytic bacteria of potato tubers (Bakken et al., 1997; Ellis et al., 2003).

According to the result of the antimicrobial test, nine strains harbored at least one positive antimicrobial activity among the 37 species, but only three strains showed the antimicrobial activity against both the two indicator pathogens, including P-HV18 (*S. kummerowiae* CCBAU 25048^T^, 98.74%), P-NA2-14 (*B. megaterium* NBRC 15308^T^, 99.31%), and P-GP5-2 (*Rhodococcus qingshengii* JCM 15477^T^, 100%). Previous studies have demonstrated that *Bacillus* strains and *Rhodococcus* strains of other plants exhibited antibacterial activity (Stein, 2005; Borisova, 2011; Radhakrishnan et al., 2017). In particular, the genus *Bacillus* is well known for the natural production of secondary metabolites with antimicrobial activities and has a very strong biocontrol potential, which not only works against phytopathogens such as *Burkholderia solanacearum* and *Fusarium oxysporum* (Yang et al., 2002; Egamberdieva et al., 2017), but also the *S. scabies* used in this study (Singh and Chaudhari, 2012). However, the antimicrobial activity of *Shinella* has rarely been reported. The strain P-HV18 (*S. kummerowiae* CCBAU 25048T, 98.74%) in this study could be used as a new active strain for the study of the antimicrobial activity of endophytic strain.

Previous studies have demonstrated that the metabolites and physiological states of a host plant can be affected by some endophytes directly or indirectly in a variety of ways (Strobel and Long, 1998; Etesami et al., 2015; Elshaghabee et al., 2017; Radhakrishnan et al., 2017). In this study, a significant fraction of the endophytic bacteria (62.16%) displayed the effect of inhibiting solanine. Among the 37 species, 23 strains showed inhibitory activity on solanine in potato tubers, while 15 strains had better inhibition on solanine in potato tubers than CIPC (the inhibition rate was 10.52%). Among them, strain P-NA2-14 (the inhibition rate was 38.37%) showed the highest inhibition on the content of solanine. There were very few previous studies on the solanine-degrading bacteria. A method for screening bacteria capable of degrading solanine was successfully developed by Hennessy et al. (2018), and 14 species of bacteria were isolated from potato soil extracts. The results revealed that most of the efficient solanine-degrading strains belonged to the genus *Arthrobacter* (Hennessy et al., 2018). The other study showed that *Bacillus subtilis* could regulate the solanine content in healthy and *Fusarium*-infected tubers of potatoes during storage (Lastochkina et al., 2020). However, the mechanisms of endophytic bacteria regulated the content of solanine were still unclear, which needed further research. It can be seen that the endophytic bacteria could be used as a safe and efficient new technology or product for the degradation of solanine, and biological control will be a promising way to reduce solanine content.

Strain P-NA2-14 (*B. megaterium* NBRC 15308^T^, 99.31%) showed not only a better antimicrobial activity against the two indicator pathogens, but also the best inhibitory activity on solanine, which was proved to be a potential biocontrol bacterium and was selected for further study. Potato tubers with low and high solanine content were obtained by spraying potato tubers with different active components of the fermentation broth of strain P-NA2-14 to study the relationship between the distribution of endophytic bacterial community and the content of solanine in potato tubers.

The results showed obvious differences in the endophytic bacteria community composition among potato tubers with different solanine contents. *Pantoea* showed the highest proportion in Group CK, *Bacillus* and *Rhodococcus* showed the highest proportion in Group H. In addition, *Myroides* and *Polaromonas* were only detected in Group L (Table 4). As the promising endophytic bacteria genera for agricultural practices, *Pantoea* and *Bacillus* have been extensively studied, which have the ability to dissolve inorganic phosphates, produce siderophores, inhibit the growth of plant pathogens, and produce indole-3-acetic acid (IAA) among others (Elshaghabee et al., 2017; Soutar and Stavrinides, 2018; Ferreira et al., 2019). The members of *Rhodococcus* are diverse catalysts that degrade a variety of both natural, organic, and xenobiotic compounds. While, some reports have shown that both *Rhodococcus* and *Polaromonas* can promote the transport of sulfur in plants (Borisova, 2011; Gahan and Schmalenberger, 2014; Kim et al., 2018). The application of *Myroides* in agriculture was rarely reported. Therefore, the isolation and identification of rare endophytic bacteria which were detected in Illumina-based analysis should be carried out by improving the culture conditions and culture techniques, and the interaction between rare endophytic bacteria and host plant metabolites should be studied further.

Meanwhile, Group L had the highest endophytic bacterial community abundance and harbored the largest number of unique OTUs, while Group CK had the least endophytic bacterial community abundance, indicating that the content of solanine influences the endophytic community composition in potato tubers (Figures 6, 8B). This was confirmed by the significant separation of the three groups on the results of PCA (Figure 9A). There was no positive correlation between the content of solanine and community composition of endophytic bacteria in potato tubers, which showed that although the content of solanine was a factor to shape the community composition of endophytic bacteria, the active ingredients of group 40–20 (inhibition rates of solanine was 41.82%) and group 20–30 (inhibition rates of solanine was −22.32%) also had some influence on the community composition of endophytic bacteria. While, the endophytic community of Group H and Group L clustered obviously and the endophytic community of Group CK was significantly different from the other two groups (Figure 9B), which further showed that both the active ingredients played an important role in shaping the community composition of endophytic bacteria. However, whether the active ingredients influenced directly on endophytic bacteria composition and then resulted in the change of solanine content, or stimulated directly on the key enzyme genes in the solanine synthesis pathway and then led to changes of community composition of endophytic bacteria is unknown, which should be the main content for illuminating the mechanism of the active ingredients (group 40–20 and group 20–30) for further study. To the best of our knowledge, this is the first report describing the relationship between the distribution of endophytic bacterial community and the content of solanine in potato tubers by Illumina-based analysis.

There is a huge potential to use plant beneficial endophytic bacteria as biofertilizers and biopesticides. Although many such bacteria have been isolated and identified, the complex dynamics that control the plant-endophyte association remains poorly understood. So, we need to identify the subtleties that govern the plant-endophyte relationship at the molecular level. The relationship between the distribution of endophytic bacterial community and the content of solanine in potato tubers was preliminarily explored in this study. Further research is needed to explore the interaction between endophytic community composition and the content of solanine by techniques like RNA-seq and Multi-group analysis technique.

## Data Availability Statement

The original contributions generated for this study are publicly available. This data can be found here: https://www.ncbi.nlm.nih.gov/sra/?term=PRJNA641941.

## Author Contributions

F-ZW, BF, and J-ML contributed to the conception and design of the study. Y-TH and JL participated in plant collection. J-ML, S-SW, XZ, and NJ performed the experiments. J-ML and S-SW performed the statistical analysis. J-ML and S-SW wrote the manuscript. XZ made a great contribution in the process of revising the manuscript. All authors approved the submitted version.

## Conflict of Interest

The authors declare that the research was conducted in the absence of any commercial or financial relationships that could be construed as a potential conflict of interest.

## Footnotes

1 http://www.fao.org/faostat/en/#data/QC
