# Supplementary material for: Antimicrobial Activity Against Phytopathogens and Inhibitory Activity on Solanine in Potatoes of the Endophytic Bacteria Isolated From Potato Tubers
Source: Front Microbiol. 2020 Nov 17;11:570926. doi: 10.3389/fmicb.2020.570926 (PMC7705204; doi:10.3389/fmicb.2020.570926)
Supplement: Supplementary file 1 [file Data_Sheet_1.PDF]

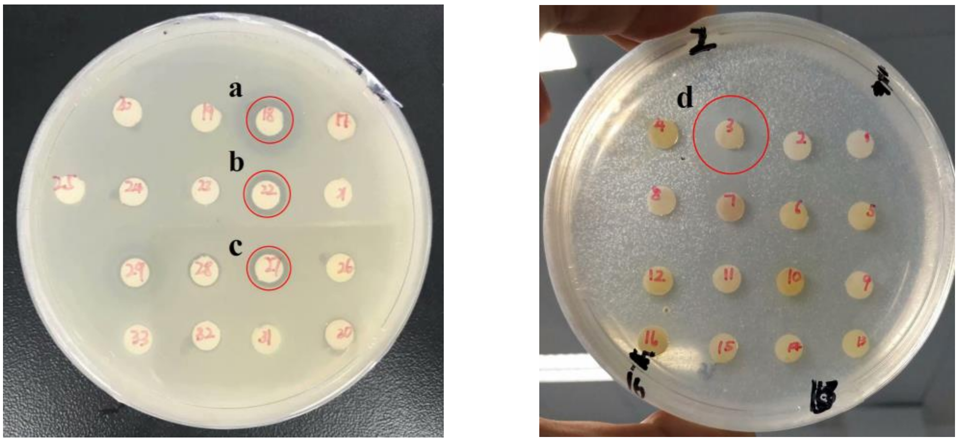

Supplementary Figure 1. Antimicrobial activity of some strains against phytopathogens. (a-Antimicrobial activity of P-TW21 against *Erwinia carotovora* subsp. *atroseptica* (Van Hall) Dye; b-Antimicrobial activity of P-GP2-2 against *Erwinia carotovora* subsp. *atroseptica* (Van Hall) Dye; c-Antimicrobial activity of P-HV18 against *Erwinia carotovora* subsp. *atroseptica* (Van Hall) Dye; d-Antimicrobial activity of P-GP5-2 against *Streptomyces scabies*.)

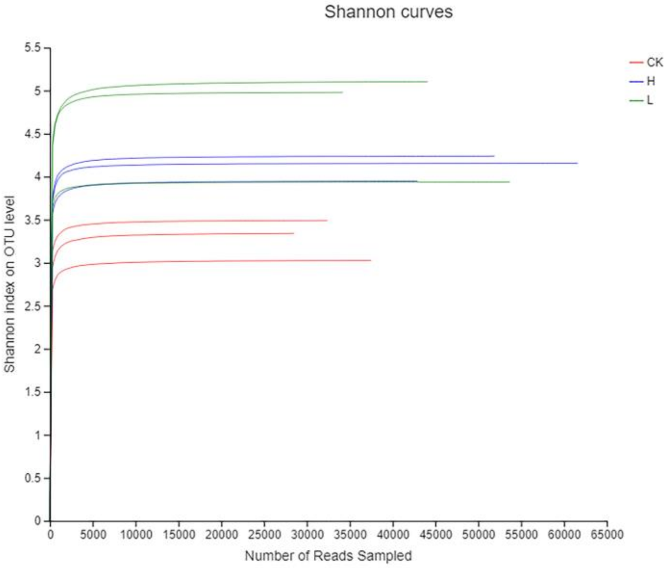

Supplementary Figure 2. Rarefaction curves of the OTU number at 97% similarity for different samples. (The rarefaction curves showed that the sequencing work was relatively comprehensive in covering the bacterial diversity, as the curves tended to approach saturation, indicating that the selected sequence data adequately reflected the bacterial abundance of these samples. Group CK: The unpeeled potato tubers were treated with blank medium, the content of solanine was 273 mg/kg; Group L: The unpeeled potato tubers were treated with the active ingredient 40-20, the content of solanine was 163 mg/kg; Group H: The unpeeled potato tubers were treated with the active ingredient 20-30, the content of solanine was 344 mg/kg.)

Supplementary Table 1. 11 different media and medium component used in this study.

**Medium Name**	**Medium Component**
Humic acid agar (HV)	$CaCO_3$ 0.02 g, Humic acid 1.0 g, KCl 1.7 g, $FeSO_4 \cdot 7H_2O$ 0.01 g, $MgSO_4 \cdot 7H_2O$ 0.05 g,

	Na_2HPO_4 0.5 g, Agar 20.0 g, ultrapure water 1 L, PH:7.2
10% YIM38 agar (YIM38)	Glucose 0.4 g, Yeast extract 0.4 g, Malt extract 0.5 g, B-Vitamins 0.1 mL / L, Trace salt 0.1 mL / L, Agar 20.0 g, ultrapure water 1 L, PH:7.2
Tap water yeast glucose starch agar (TW)	Yeast extract 0.25 g, K_2HPO_4 0.5 g, Agar 20.0 g, Tap water 1 L, PH:7.2
Glucose starch agar (BL)	Glucose 5.0 g, Yeast extract 5.0 g, Soluble starch 5.0 g, Casamino acids hydrolysate 2.0 g, $CaCO_3$ 5.0 g, NaCl 5.0 g, Agar 20.0 g, ultrapure water 1 L, PH:7.2
Sodium propionate agar (SP)	$CaCl_2$•$2H_2O$ 0.02g, Sodium propionate 1.0 g, L-Asparagine 0.2 g, K_2HPO_4 0.6 g, KH_2PO_4 0.9 g, $MgSO_4$•$7H_2O$ 0.1 g, Agar 20.0 g, ultrapure water 1 L, PH:7.2
Trehalose – proline agar (TP)	$CaCl_2$ 2.0 g, $(NH_4)_2SO_4$ 1.0 g, Trehalose 5.0 g, Proline1.0 g, NaCl 1.0 g, K_2HPO_4 1.0 g, $MgSO_4$•$7H_2O$ 1.0 g, B-Vitamins 1 mL, Agar 20.0 g, ultrapure water 1 L, PH:7.2
Cellulose agar (CM)	$CaCO_3$ 0.02 g, KNO_3 0.2 g, Cellulose 10.0 g, Casein 0.3 g, K_2HPO_4 0.2 g, $FeSO_4$ 0.01 g, $MgSO_4$•$7H_2O$ 0.05 g, Agar 20.0 g, ultrapure water 1 L, PH:7.2
M-WA agar (GP)	Glycerol 10.0 g, Yeast extract 0.5 g, KNO_3 0.5 g, Proline1.0 g, L-Asparagine 1.0 g, Sodium pyruvate 1.25 g, Betaine 1.25 g, Agar 20.0 g, ultrapure water 1 L, PH:7.2
10% Nutrient agar (NA)	Beef extract 0.5 g, Peptone 1 g, Sodium pyruvate 1.25 g, Betaine 1.25 g, NaCl 10 g, Agar 20.0 g, ultrapure water 1 L, PH:7.2
Raffinose–histidine agar (RH)	Raffinose 1.0 g, $CaCO_3$ 0.02 g, Histidine 0.1 g, Na_2HPO_4 0.5 g, KCl 1.7 g, $MgSO_4$•$7H_2O$ 0.05 g, $FeSO_4$•$7H_2O$ 0.1 g, B-Viramins 1 mL, Agar 20.0 g, ultrapure water 1 L, PH:7.2
R2A agar (R2A)	Glucose 0.5 g, Yeast extract 0.5 g, Peptone 0.5 g, Casein peptone 0.5g, Sodium pyruvate 0.3g, $MgSO_4$•$7H_2O$ 0.024 g, K_2HPO_4 0.3 g, Soluble starch 0.5g, Agar 20.0 g, ultrapure water 1 L, PH:7.2

Supplementary Table 2. PCR amplification procedure.

**Reaction procedure**	**Temperature/°C**	**Time**	
Pre-degeneration	95	2min	
Denaturation	95	30s	
Anneal	72	30s	25 cycles
Extension	72	3s	
Final extension	72	5min	

PCR reactions were performed in triplicate 20 μL mixture containing 4 μL of 5 × FastPfu Buffer, 2 μL of 2.5 mM dNTPs, 0.8 μL of each primer (5 μM), 0.4 μL of FastPfu Polymerase, and 10 ng of template DNA.
